# Adaptive noise-augmented attention for enhancing Transformer fine-tuning on longitudinal medical data

**DOI:** 10.3389/frai.2025.1663484

**Published:** 2025-09-17

**Authors:** Ali Amirahmadi, Farzaneh Etminani, Mattias Ohlsson

**Affiliations:** ^1^Center for Applied Intelligent Systems Research in Health, Halmstad University, Halmstad, Sweden; ^2^Department of Research and Development (FoU), Region Halland, Halmstad, Sweden; ^3^Centre for Environmental and Climate Science, Computational Science for Health and Environment, Lund University, Lund, Sweden

**Keywords:** Transformer, augmentation, adaptive noise, medical data, electronic health records (EHR), fine-tuning, representation learning, self-attention

## Abstract

Transformer models pre-trained on self-supervised tasks and fine-tuned on downstream objectives have achieved remarkable results across a variety of domains. However, fine-tuning these models for clinical predictions from longitudinal medical data, such as electronic health records (EHR), remains challenging due to limited labeled data and the complex, event-driven nature of medical sequences. While self-attention mechanisms are powerful for capturing relationships within sequences, they may underperform when modeling subtle dependencies between sparse clinical events under limited supervision. We introduce a simple yet effective fine-tuning technique, Adaptive Noise-Augmented Attention (ANAA), which injects adaptive noise directly into the self-attention weights and applies a 2D Gaussian kernel to smooth the resulting attention maps. This mechanism broadens the attention distribution across tokens while refining it to emphasize more informative events. Unlike prior approaches that require expensive modifications to the architecture and pre-training phase, ANAA operates entirely during fine-tuning. Empirical results across multiple clinical prediction tasks demonstrate consistent performance improvements. Furthermore, we analyze how ANAA shapes the learned attention behavior, offering interpretable insights into the model's handling of temporal dependencies in EHR data.

## 1 Introduction

Foundation models, deep neural networks pre-trained on broad unlabeled data using self-supervised methods, have significantly impacted various aspects of our lives, including law, healthcare, education, and more (Bommasani et al., 2021; Guo et al., 2023; Wornow et al., 2023). These models typically acquire general knowledge about the data through pre-training a variant of the Transformer network on a self-supervised task like Masked Language Model (MLM), and then adapt this knowledge to downstream tasks with only a few labeled samples during the fine-tuning process. Researchers showed that pre-training, even with limited data, can improve Transformers' performance significantly (Amos et al., 2023).

Pre-training Transformers have been employed with various self-supervised objectives and domains. Common objectives include corrupted text reconstruction tasks like MLM (Devlin et al., 2018; Lewis et al., 2019; Lan et al., 2019) and standard language models such as next-word prediction (Radford et al., 2019; Brown et al., 2020), which have been extensively utilized (Liu et al., 2023). These models typically adopt a backbone architecture inspired by the multi-head attention mechanism in Transformers (Vaswani et al., 2017), known for its effectiveness in modeling complex interaction between events (tokens) in a sequence (text). These foundation models have been pre-trained on different domain data (Lan et al., 2019; Radford et al., 2019), including structured temporal health data as sequences of events (Li et al., 2020; Rasmy et al., 2021; Pang et al., 2021).

Modeling Electronic Health Records (EHRs) trajectories presents a critical opportunity for predicting health-related outcomes, offering benefits like early intervention, cost reduction, and improved public health. This field has attracted significant attention from deep learning researchers (Xiao et al., 2018; Amirahmadi et al., 2023; Boll et al., 2024; Li et al., 2024). Typically, healthcare specific foundation models are pre-trained on publicly available, unlabeled EHR data, and adapting these models through fine-tuning consistently demonstrates superior performance across various tasks (Li et al., 2020; Rasmy et al., 2021; Pang et al., 2021; Ren et al., 2021; Li et al., 2022; Yuanyuan et al., 2025).

**Figure 1:**
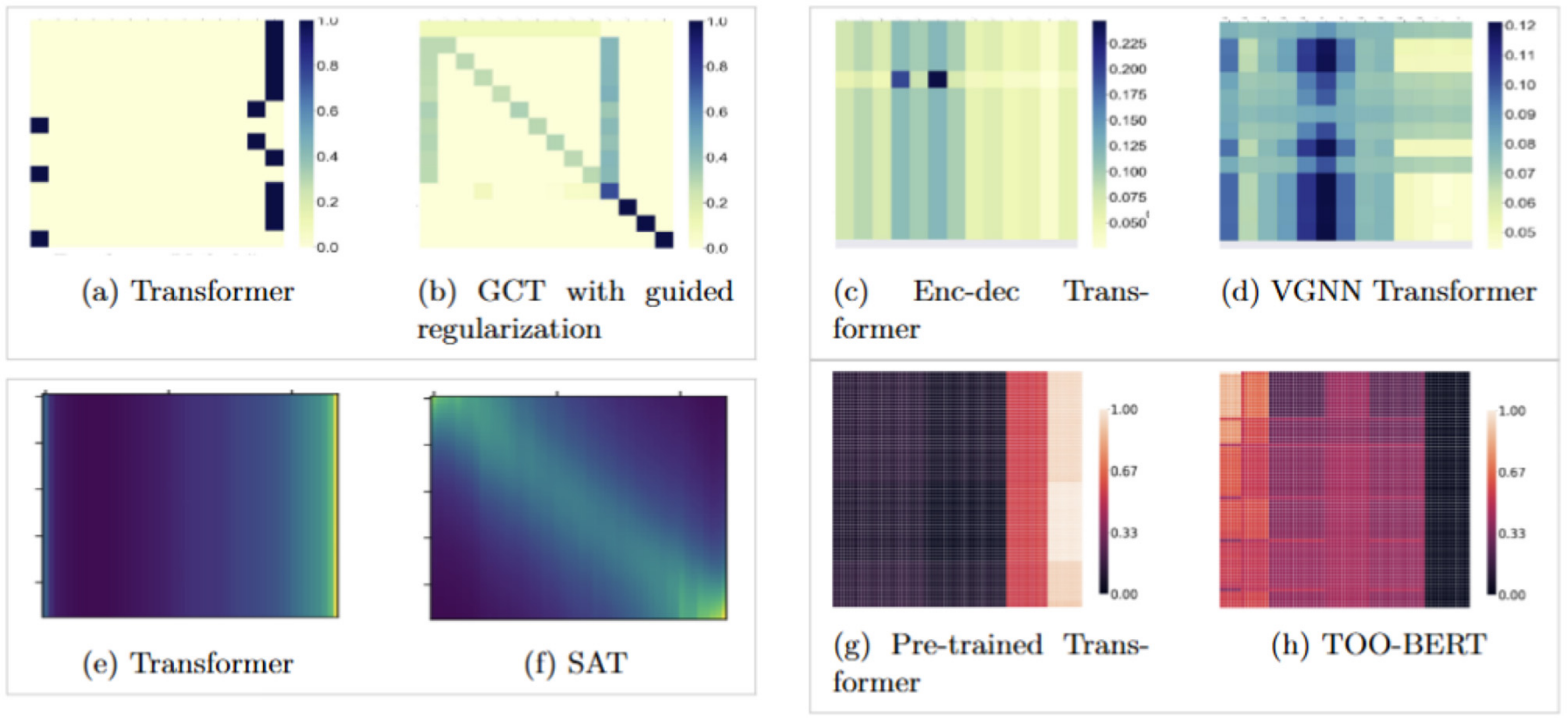
Visualization of attention score patterns for different models from previous studies and how their proposed methods helping a more complicated structure in attention scores in Transformers. **(a, b)** Transformer trained from random weights vs. Transformer trained with domain knowledge (Zhu and Razavian, 2021; Choi et al., 2020). **(c, d)** Encoder-decoder vs. VGNN using variational regularization (Zhu and Razavian, 2021). **(e, f)** Vanilla Transformer vs. SAT with temporal priors (Kim and Lee, 2024). **(g, h)** Transformer pre-trained on MLM vs. MLM with trajectory order prediction (Amirahmadi et al., 2025). **(e, f)** Had no color bars in the original papers.

However, EHRs are often scarce, and training Transformers to learn the complex relationships between medical events in longitudinal EHRs requires either large amounts of data, or advanced training techniques and augmentations (Dosovitskiy et al., 2020; Touvron et al., 2021; Hassani et al., 2021, 2023). Due to privacy concerns and the scarcity of publicly available datasets, models often fail to learn the intricate dependencies between events in a patient's history. To address this, (Choi et al. 2020) proposed incorporating domain knowledge into the attention mechanism, while (Zhu and Razavian 2021) employed variational regularization. Additionally, (Amirahmadi et al. 2025) suggested pre-training the Transformer on the MLM task and the ordering of medical events in a patient's history, and (Kim and Lee 2024) proposed using learnable, adaptive kernels in the attention matrices to improve contextual representations and enhance the learned structure through self-attention. Figures 1, 4 illustrate how these various approaches impact self-attention behaviors in leaning the relationships between events. However, these methods often come with substantial computational costs and require extra effort for implementation and design.

Data augmentation is another solution to tackle the data scarcity challenge. Augmenting data with discrete data types, such as series of medical codes or tokens in text, is challenging because small perturbations can drastically alter semantic meaning, and interpolation in discrete space is not feasible (Chen et al., 2020). For example, replacing a code for “Type 1 diabetes” with “Type 2 diabetes,” or reordering diagnosis and procedure codes within the same patient trajectory, can fundamentally change the clinical context. As a result, researchers have proposed augmenting models during training as an alternative (Jain et al., 2023; Zehui et al., 2019; Wu et al., 2023).

In this study, we propose a simple two-step augmentation technique-Adaptive Noise-Augmented Attention (ANAA)—that perturbs attention scores by injecting adaptive Gaussian noise followed by smoothing with a Gaussian kernel. Our investigation of attention distributions reveals that fine-tuned Transformers tend to produce highly polarized attention scores—values clustering near the extremes (0 or 1), which restricts the model's capacity to explore diverse dependencies (see the bottom row of **Figure 4**). By introducing controlled noise into attention scores during fine-tuning, we encourage exploration of alternative dependency paths between events. The subsequent smoothing operation helps restore structural consistency while preserving diversity, resulting in more balanced and informative self-attention maps.

The main contributions are summarized as follows:

1. We proposed a simple self-attention augmentation method that encourages the model to explore and learn more complex attention patterns during fine-tuning. Importantly, this approach does not modify the computational graph, making it easily applicable to any pre-trained Transformer.
2. We conducted several evaluations on various downstream tasks, examining the effect of the novel method on model performance, model robustness with limited training samples, and the balance of attention distribution between distant and nearby events. Our results demonstrate how it improves the performance of pre-trained Transformers.

## 2 Preliminary

### 2.1 Transformer encoder and self-attention

The core back-bone of Transformers encoder is the multi-head self-attention. Each self-attention head is:


(1) $$\begin{array}{l} Q_{h}=XW_{h}^{Q},K_{h}=XW_{h}^{K},V_{h}=XW_{h}^{V}, \end{array}$$

(2) $$\begin{array}{l} A_{h}=\text{softmax}\left(\frac{Q_{h}K_{h}^{T}}{\sqrt{d_{k}}}\right) \end{array}$$

(3) $$\begin{array}{l} H_{h}=\text{Self-attention}\left(X\right)=A_{h}V_{h} \end{array}$$

Where, $Q,K\in {\mathbb{R}}^{n\times d_{k}}$ and $V\in {\mathbb{R}}^{n\times d_{v}}$ and *n* is the length of input sequence and *d*_*k*_ and *d*_*v*_ are dimenssion of Key and Value. *A*_*h*_ is the attention score matrix and each *A*_*i, j*_ indicates how much attention token *x*_*i*_ put on *x*_*j*_. Transformer encoders, is built on concatenation of ∣*h*∣ number attention heads in parallel, so each one has its own weights. Then, the concatenation is projected:


(4) $$\begin{array}{l} \text{MultiHead}\left(X\right)=\text{Concat}\left(H_{1}....,H_{|h|}\right)W^{O} \end{array}$$

Where, $W^{O}\in {\mathbb{R}}^{|h|\times d_{v}}$ Multiple self-attention heads in parallel, help the model to attend to information from different representation subspaces (Vaswani et al., 2017; Hao et al., 2021).

### 2.2 Pre-training, fine-tuning

Pretraining typically involves the model acquiring general knowledge, which is then used to initialize the final network. Subsequently, the final network adjusts these weights to obtain optimized weights for specific downstream tasks (Chen et al., 2021). This approach has been extensively utilized for adapting foundation models to downstream tasks (Lan et al., 2019; Liu et al., 2023).

## 3 Related works

Advanced training techniques and data augmentation have been widely adopted to improve the performance of Transformer models, especially in settings with limited labeled data. These methods aim to enhance the generalizability and robustness of learned representations.

Several methods modify self-attention to better learn intricate local and global attentions between different tokens. (Hassani et al. 2023) introduced a sliding window attention mechanism to localize attention spans and improve efficiency. (Ding et al. 2023) reduced attention complexity by segmenting key, query, and value inputs and sparsifying their interactions, allowing Transformers to better model both short- and long-range dependencies. Positional encoding has also been a target for improvement: (Su et al. 2024) and Press et al. (2021) enhanced distant token interaction by encoding absolute positions with rotation matrices or distance-based penalties on query-key attention scores. While these methods are effective, they often require structural changes to the attention mechanism, making them less compatible with pre-trained models and harder to integrate into existing pipelines.

Data augmentation is another solution to tackle the data scarcity challenge, but it is particularly challenging in discrete domains like medical codes or text, where small changes can drastically alter semantic meaning and interpolation is not well-defined (Chen et al., 2020). To address this, researchers have proposed augmenting models during training or fine-tuning by injecting noise into internal representations (Jain et al., 2023; Zehui et al., 2019; Yuan et al., 2022; Wornow et al., 2023; Wu et al., 2023). Injecting Gaussian noise into activations has been shown to help models converge to smoother minima, improving generalization, calibration, and robustness to perturbations (Camuto et al., 2020). (Zhu et al. 2019) enhanced the performance of BERT (Devlin et al., 2018) and RoBERTa (Liu et al., 2019) by adding adversarial noise to word embeddings, a technique later extended to graph neural networks by (Kong et al. 2022) for improved out-of-distribution generalization. In the self-attention space, (Zehui et al. 2019) proposed DropAttention, which randomly masks and expands attention scores to regularize focus. Similarly, (Wu et al. 2023) introduced adversarial structural biases to attention matrices, though at the cost of increased training complexity.

(Wornow et al. 2023) injected Gaussian noise into the latent space of an encoder-decoder model for better image captioning, while (Yuan et al. 2022) perturbed hidden representations during fine-tuning to marginally improve language model performance. Most notably, (Jain et al. 2023) introduced NEFTune, which adds calibrated uniform noise to embedding vectors during fine-tuning—resulting in significant improvements for models like LLaMA-1 and LLaMA-2. Inspired by these efforts, we compare our method with NEFTune and propose a new approach that directly perturbs the attention scores, encouraging the model to learn richer contextual dependencies across sequences. Here, We investigate augmenting the self-attention scores—central to modeling event dependencies—by injecting and smoothing adaptive Gaussian noise. Unlike prior methods that perturb embeddings or hidden states, our approach directly improves attention behavior without changing the model architecture, enhancing the learned representation in a lightweight and effective way.

## 4 Methods

### 4.1 Adaptive noise-augmented attention

In this subsection, we introduce, Adaptive Noise-Augmented Attention (ANAA), a simple yet effective two-step augmentation technique designed to improve the learned representations in Transformer models by directly augmenting the attention scores during fine-tuning (Algorithm 1). This method enhances attention dynamics without modifying the computational graph, making it compatible with any pre-trained Transformer encoder.

**Algorithm 1:** Fine-tuning Transformer encoder with ANAA.

**Input**: $D_{\text{fine-tuning}}={\left\{\left(X_{i},y_{i}\right)\right\}}_{1}^{N}$ tokenized dataset, embedding layer emb(·), attention score matrix *A*_*h*_, normal noise $N\left(\mu ,\sigma_{\text{GN}}^{2}\right)$, two-dimensional Gaussian noise *n*_σ_eh__, rest of the model *f*(·)
**Parameter**: Normal noise $\mu ,\sigma_{\text{GN}}^{2}$ calculated from *A*_*h*_, event horizon hyperparameter σ_eh_ based on the data charecterstic needs to adjust the smoothing noise
1Initialize θ from a pre-trained model 2**repeat**
3 Sample (*X*_*i*_, *y*_*i*_)~*D*_fine-tuning_ *X*_emb_←emb(*X*_*i*_)
**for** each Attention Head *A*_*h*_ in Transformer Block
**do** $A_{h}\left(X_{\text{attn}}\right)\leftarrow A_{h}\left(X_{\text{emb}}\right)+N\left(\mu ,\sigma_{\text{GN}}^{2}\right)$
*A*_*h*_(*X*_attn_)←Convolve(*A*_*h*_(*X*_attn_), *n*_σ_eh__) *H*_*h*_(*X*_attn_)←*A*_*h*_(*X*_attn_)*V* **end for** MultiHead(*H*)←concat(*H*_0_(*X*_attn_), …, *H*_*h*_(*X*_attn_)) ŷ_*i*_←*f*(MultiHead(*H*)) θ←opt(θ, loss(ŷ_*i*_, *y*_*i*_)) **until** Stopping criteria met or maximum iterations reached

ANAA operates by first injecting adaptive Gaussian noise into the attention score matrix and then applying a smoothing operation using a Gaussian kernel. This process encourages the model to explore the attention patterns and strengthens context modeling. The augmented attention is computed as:


(5) $$\begin{array}{l} \text{ANAA}=\left(\left(A_{h}+\sim N\left(\mu ,\sigma_{GN}^{2}\right)\right)*n_{\sigma_{eh}}\right)V \end{array}$$

Here, the Gaussian noise $N\left(\mu ,\sigma_{\text{GN}}^{2}\right)$ is computed adaptivly based on the learned attention during training:


(6) $$\begin{array}{ll} \mu & =\frac{1}{n^{2}}\overset{n-1}{\underset{i=0}{\sum }}\overset{n-1}{\underset{j=0}{\sum }}A_{i,j} \end{array}$$

(7) $$\begin{array}{ll} \sigma_{\text{GN}} & =\sqrt{\frac{1}{n-1}\overset{n-1}{\underset{i=0}{\sum }}\overset{n-1}{\underset{j=0}{\sum }}{\left(A_{i,j}-\mu \right)}^{2}} \end{array}$$

The smoothing kernel *n*_σ_eh__[*i, j*] is a 2D Gaussian distribution:


(8) $$\begin{array}{l} n_{\sigma_{\text{eh}}}\left[i,j\right]=\frac{1}{2\pi \sigma_{\text{eh}}^{2}}e^{-\frac{1}{2}\left(\frac{i^{2}+j^{2}}{\sigma_{\text{eh}}^{2}}\right)} \end{array}$$

where σ_eh_ is a tunable hyperparameter representing the event horizon, controlling and adjusting the extent of the smoothing. The convolution operation * applies this kernel over the noise-augmented attention matrix:


(9) $$\begin{array}{l} f\left[i,j\right]*n_{\sigma }\left[i,j\right]=\frac{1}{2\pi \sigma^{2}}\overset{k}{\underset{m=1}{\sum }}\overset{k}{\underset{n=1}{\sum }}e^{-\frac{1}{2}\left(\frac{m^{2}+n^{2}}{\sigma^{2}}\right)}f\left[i-m,j-n\right] \end{array}$$

where *k* = 2πσ is the kernel size.

**Figure 2:**
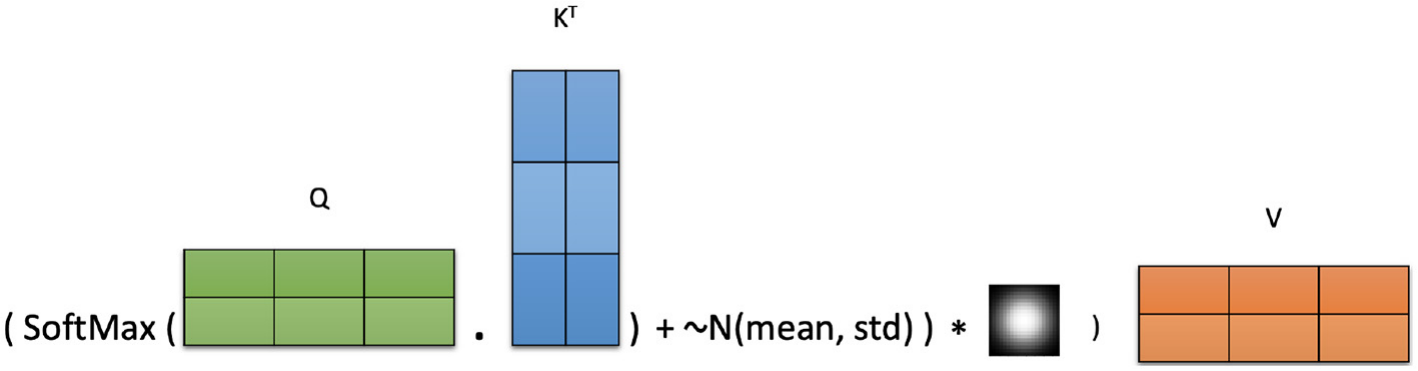
Adaptive Noise-Augmented Attention (ANAA) mechanism.

This smoothing step modulates the added noise, reinforcing stronger attention patterns while allowing for broader exploration in attentions space. The noise parameters μ and σ_GN_ are computed independently for each attention head to preserve head-specific attention dynamics during training. Figure 2 illustrates the full ANAA mechanism.

Adding adaptive Gaussian noise $\sim N\left(\mu ,\sigma_{GN}^{2}\right)$ to the attention scores helps the model escape sub-optimal solutions and promotes learning more diverse interactions between events. The subsequent Gaussian convolution adjusts the magnitude and distribution of the injected noise, encouraging the model to focus on more meaningful and effective attention patterns.

During inference, stochasticity from the added noise is removed by replacing it with its expected value μ, ensuring deterministic predictions:


(10) $$\begin{array}{l} \text{ANAA}=\left(\left(A_{h}+\mu \right)*n_{\sigma_{eh}}\right)V \end{array}$$

The computational complexity of ANAA is *O*(*n*^2^) (for more details, see the Supplementary material Section 1.9, and since it's primarily used during fine-tuning with limited labeled samples, the additional cost is negligible.

### 4.2 Mechanistic rationale: Why ANAA works

**Figure 4** reveals that, in the absence of augmentation, many attention heads converge to a degenerate two-point distribution: each weight is either exactly 0 (“off”) or 1 (“on”), with masses 1−α and α, respectively. ANAA first perturbs the Attention scores with adaptive Gaussian noise whose variance scales as α(1−α) (Supplementary material Section 1.5). So, every Dirac spike is broadened into a narrow normal curve, turning the rigid on/off pattern into a bimodal continuous distribution that expresses graded less-important vs. more-important scores. The subsequent Gaussian convolution (Supplementary material Section 1.6) behaves as a data-adaptive low-pass filter: it suppresses high-frequency artifacts and interpolates between neighboring tokens, so the random, isolated spikes introduced by the noise disappear.

Taken together, ANAA can be viewed as a variance-scaled, structured drop-connect regularizer (Supplementary material Section 1.7), analogous to–but more principled than–classical dropout, which disconnects token pairs with an independent Bernoulli mask. ANAA instead perturbs each attention score additively, so every mini-batch sees a different, spatially smoothed view of the inputs relations.

## 5 Experiments

### 5.1 Datasets

In our study, we utilized medical data from two sources: the MIMIC-IV (Johnson et al., 2020) hosp module and the Malmö Diet and Cancer Cohort (MDC) (Berglund et al., 1993) dataset, approved by the Ethics Review Board of Sweden (Dnr 2023-00503-01). Each EHR trajectory represents a sequence of temporally structured health events. The MIMIC-IV dataset includes 173,000 patient records across 407,000 visits from 2008 to 2019, with 10.6 million medical codes. The MDC dataset, from a cohort study in Sweden, comprises 30,000 individuals with 531,000 visits from 1992 to 2020, offering a more extended patient history—257 codes per patient on average, compared to MIMIC-IV's 61. To ensure consistency, we used only ICD and ATC codes, the only types available in MDC at the beginning, aligning with prior work like Med-BERT on diagnosis codes for risk prediction.

Both datasets use ICD and ATC codes for disease and medication classification. We randomly split each cohort into 70% for pre-training, 20% for fine-tuning, and 10% for testing. After preprocessing, MIMIC-IV had 2,195 unique ICD-9 and 137 ATC-5 codes, while MDC had 1,558 ICD-10 and 111 ATC-5 codes. To assess the generalizability and robustness of our results, the fine-tuning dataset was split into 5 folds. The model was fine-tuned on 4 folds with early stopping on the remaining fold, repeated 5 times with different validation sets. We reported the mean and standard deviation of the AUC on the unseen test dataset. For details, refer to the dataset availability, specifications and implementation details in the Supplementary material Sections 1.1, 1.2, 1.4.

### 5.2 Problem formulation

Each dataset *D* comprises a set of patients *P*, *D* = {*P*^1^, *P*^2^, …, *P*^|*D*|^}. In our study, we considered a total of |*D*| = 172, 980 patients for MIMIC-IV and |*D*| = 29, 664 patients for the MDC cohort. We represent each patient's longitudinal medical trajectory through a structured set of visit encounters as a sequence of events. This representation is denoted as $P^{i}=\left\{V_{1}^{i},V_{2}^{i},\ldots ,V_{O}^{i}\right\}$, where *O* represents the total number of visit encounters for patient *i*. Each visit $V_{j}^{i}=I_{j}\cup M_{j}$ is the union of all diagnosis codes *I*_*j*_⊂*I* and prescribed medications *M*_*j*_⊂*M* that are recorded for the *P*^*i*^ at visit $V_{j}^{i}$. To reduce sparsity, we excluded less frequently occurring medical codes and retained only the initial 4 digits of ICD and ATC codes.

To guide the model in understanding changes in encounter times and the structure of each patient's trajectory, similar to BERT, we employed special tokens. A [*CLS*] token is placed at the beginning of each patient's trajectory, while a [*SEP*] token is inserted between visits. Each visit represents a set of diagnoses and medications recorded within a specific time span, and the [*SEP*] token separates the sets of medical codes from one visit to the next. Consequently, each patient's trajectory is represented as $P^{i}=\left\{\left[CLS\right],V_{1}^{i},\left[SEP\right],V_{2}^{i},\left[SEP\right],\ldots ,V_{O}^{i},\left[SEP\right]\right\}$, providing the model with valuable context for analysis and prediction.

Here, we evaluated our models on 3 downstream tasks *e*_*dt*_ [Heart Failure (HF), Alzheimer's Disease (AD), Prolonged Length of Stay on the next visit (PLS) predictions], where the model predicts the incidence of the first HF (*I*_*N* = *HF*_) or AD (*I*_*N* = *AD*_) ICD codes or the presence of PLS (*PLS*_*N*_ = 1) on the *N*^*th*^ visit, given the patient's previous history of medical codes, $\left[V_{1}^{i}:V_{N-1}^{i}\right]$, as a sequence of temporally structured health events:


(11) $$\begin{array}{l} \begin{array}{c} \mathbb{P}\left(e_{dt}\in V^{N}|P^{i}=\left\{\left[CLS\right],V_{1}^{i},\left[SEP\right],V_{2}^{i},\left[SEP\right],\ldots ,V_{N-1}^{i},\left[SEP\right]\right\}\right) \end{array} \end{array}$$

For each patient's trajectory, if there were no occurrences of the target events *e*_*dt*_, it is considered a negative case; otherwise, we exclude the first visit with the target and all subsequent visits and consider it a positive case. All ATC codes related to HF treatment are excluded to avoid timing-related noise and non-trivial predictions. Initially, models exhibited bias toward longer visit histories, confounding risk predictions. To address this, we excluded trajectories with fewer than 30 visits in the MDC dataset and fewer than 10 visits in the MIMIC-IV dataset. This ensured balanced visit histories between positive and negative cases, resulting in averages of 19 visits in the MDC dataset and 9 visits in the MIMIC-IV dataset, aligning with their overall dataset averages prior to preprocessing. Table 1 summarizes the number of positive and negative cases after these preprocessing steps.

**Table 1:** Number of positive and negative samples in each downstream task.

**Task**	**Positive**	**Negative**
PLS prediction	2,429	6,360
HF prediction (MIMIC-IV)	243	641
AD prediction	245	2,628
HF prediction (MDC)	103	301

### 5.3 List of models

To thoroughly investigate the impact of the proposed ANAA augmentation, we compared the performance of following conventional and deep learning models on downstream tasks of HF, AD, and PLS prediction using both the MDC and MIMIC-IV datasets. These models were trained either from scratch or initiated from pre-trained weights, fine-tuned on the fine-tuning dataset, and evaluated on the test dataset. We set the tunable event horizon parameter to σ_*eh*_ = 1.0 (kernel size = 6) for the ANAA on the MDC dataset and σ_*eh*_ = 0.33 (kernel size = 2) on the MIMIC IV after fine-tuning on the fine-tuning dataset. Except fir HF prediction in the MDC, different σ_*eh*_, slightly changes the ANAA performance. For more details see Supplementary material Section 1.3.

#### 5.3.1 Models with proposed RNA/ANAA

- **Transformer with ANAA**: This model incorporates ANAA into all self-attention heads of a randomly initialized Transformer.
- **Transformer pre-trained on MLM with Raw Noise injected Attention (RNA)**: In this approach, $N\left(\mu ,\sigma_{GN}^{2}\right)$ (normal noise with adaptive parameters) is added to all self-attention heads of a pre-trained Transformer. This experiment allows us to isolate the impact of the noise injection from the smoothing effect of Gaussian convolution.
- **Transformer pre-trained on MLM with ANAA**: This model incorporates ANAA into all self-attention heads of the pre-trained Transformer.

Baseline model details and results are provided in Supplementary material Section 1.11.

### 5.4 Evaluation on downstream tasks

The results are summarized in Table 2 and suggest that adding ANAA improves the AUC of pre-trained Transformers, potentially positioning them as one of the state-of-the-art methods for outcome prediction on temporal structured health data. Specifically, on the MDC dataset, the AUC for HF and AD prediction increased to 74.5% and 73.2%, respectively, while on the MIMIC-IV dataset, the AUC for HF prediction reached 87.2%. The addition of ANAA resulted in statistically significant improvements for HF prediction on both the MDC and MIMIC-IV datasets for the MLM pre-trained Transformer. Furthermore, the improvement in AD prediction was considerable, showcasing the effectiveness of ANAA augmentation. However, incorporating ANAA did not significantly alter the performance of PLS prediction. Additionally, applying ANAA to randomly initialized Transformers boosted the AUC for PLS prediction to 60.2%, with negligible effects on other downstream tasks. To delve deeper into the impact of each noise injection and smoothing augmentation term, we solely added the normal noise to the pre-trained Transformer. This experiment revealed that the noise injection alone had a more pronounced effect on downstream tasks in the MIMIC dataset, whereas the combined (ANAA) terms exhibited greater impacts on the downstream tasks in the MDC dataset, particularly associated with its longer sequences.

**Table 2:** Average AUC values (%) and standard deviation for different methods for the HF prediction, AD prediction, and PLS prediction downstream tasks on the test datasets.

**Model/downstream task**	**HF prediction (MDC)**	**AD prediction (MDC)**	**HF prediction (MIMIC-IV)**	**PLS prediction (MIMIC-IV)**
Transformer	71.4 (0.5)	70.5 (0.8)	84.2 (1.4)	54.4 (0.8)
Transformer+ ANAA	72.1 (2.7)	70.4 (0.6)	83.2 (2.5)	60.2 (1.2)
Transformer pre-trained on MLM	72.2 (2.5)	72.2 (1.1)	85.2 (1.1)	60.3 (1.3)
Transformer pre-trained on MLM+ RNA	72.6 (1.9)	71.4 (1.0)	86.5 (1.2)	**60.7 (0.6)**
Transformer pre-trained on MLM+ ANAA	**74.5 (2.9)**	**73.2 (0.3)**	**87.2 (0.4)**	60.3 (0.7)

Boldface indicates the best-performing model.

### 5.5 Performance boost on data insufficiency

**Figure 3:**
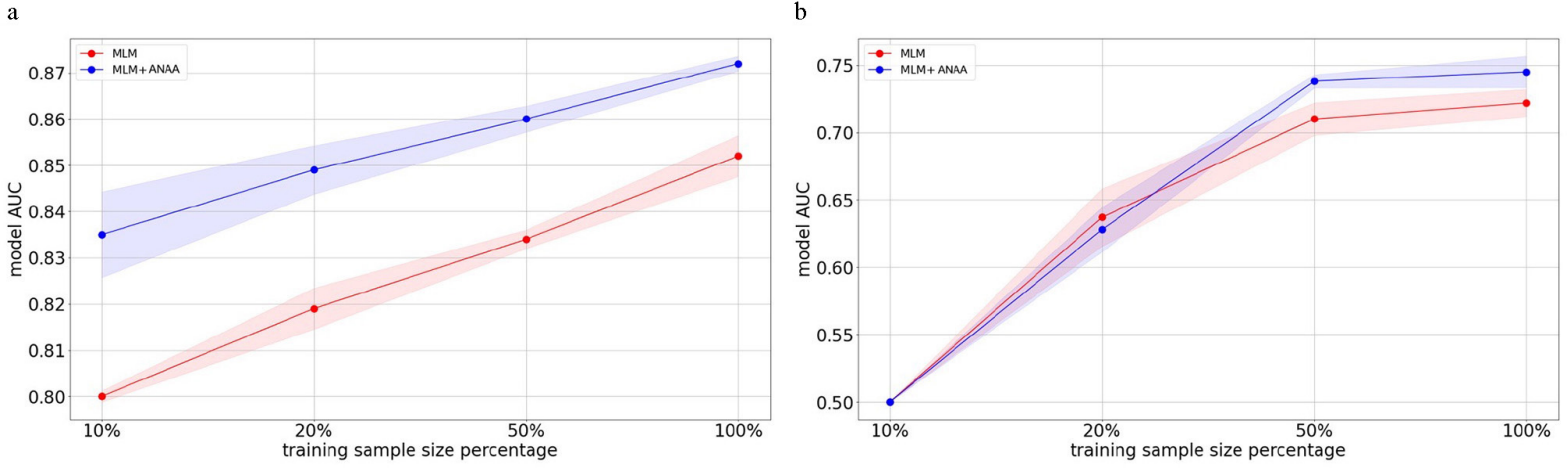
Impact of ANAA on AUC for HF prediction across different fine-tuning sample sizes in the MIMIC-IV and MDC datasets. The red line shows the AUC of a Transformer model pre-trained on MLM without augmentation; the blue line shows the AUC of the same model augmented with ANAA. In MIMIC-IV, MLM+ANAA consistently outperforms the MLM baseline at all sample sizes. In MDC, MLM+ANAA outperforms the baseline up to the 50% training size; at smaller sizes, its performance converges to that of the baseline due to the limited number of HF-positive samples in the MDC dataset. **(a)** AUC values for HF prediction across fine-tuning sample sizes on the MIMIC-IV test set. **(b)** AUC values for HF prediction across fine-tuning sample sizes on the MDC test set.

**Figure 4:**
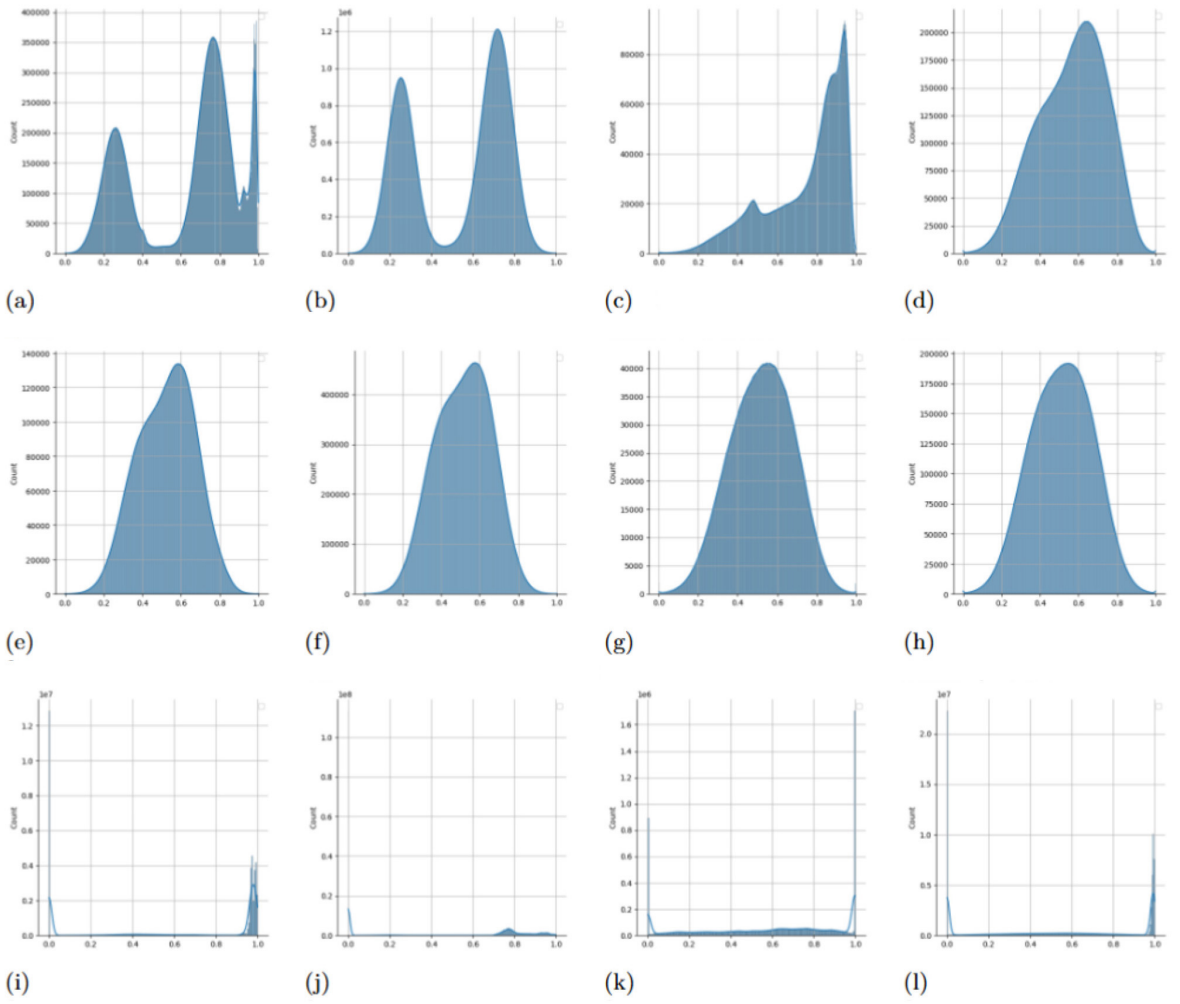
Comparison of the impact of ANAA on self-attention score distributions in fine-tuned models. Attention scores from each head are individually scaled to the [0, 1] range before plotting their distributions. **(a)** Pre-trained Transformer + Smoothed noise. **(b)** Pre-trained Transformer + ANAA. **(c)** Pre-trained Transformer + ANAA. **(d)** Pre-trained Transformer + ANAA. **(e)** Pre-trained Transformer + RNA. **(f)** Pre-trained Transformer + RNA. **(g)** Pre-trained Transformer + RNA. **(h)** Pre-trained Transformer + RNA. **(i)** Pre-trained Transformer. **(j)** Pre-trained Transformer. **(k)** Pre-trained Transformer. **(l)** Pre-trained Transformer.

One of the advantages of using pre-trained Transformers is their robustness and performance in situations of data insufficiency, observed in both NLP (Brown et al., 2020) and temporal health data (Rasmy et al., 2021). Here, we investigated the effect of applying ANAA on model performance for HF prediction with reduced data sample sizes. We decreased the fine-tuning sample size to 50%, 20%, and 10%, respectively. The performance of the pre-trained Transformer with and without ANAA, was compared on both the MDC and MIMIC-IV datasets. Figures 3a, 4 shows that ANAA improves the model performance by around 3% in HF prediction on the MIMIC-IV dataset across all data sample sizes. Similarly, Figures 3b, 4 demonstrates that ANAA consistently outperforms the baseline in HF prediction on the MDC dataset, even with a 50% reduction in training samples. However, its superiority diminishes with less data.

### 5.6 VS hidden representation augmentation

We first compared ANAA with other hidden representation augmentation methods proposed for augmenting different layers of pre-trained Transformers. Specifically, we assess the impact of injecting noise into various components of the network, such as hidden layers and feedforward modules, as explored in works like HyPe (Yuan et al., 2022) and Neftune (Jain et al., 2023). Our objective is to evaluate whether augmenting self-attention scores, where contextual dependencies are explicitly encoded, is more effective than augmenting other internal representations.

As shown in Table 3, although NefTune (Jain et al., 2023) enhances the performance of pre-trained Transformers in HF prediction across both datasets, ANAA consistently outperforms both NefTune and feedforward noise augmentation in predicting outcomes. While ANAA demonstrates superior performance in this context, NefTune has the advantage of being computationally lighter. However, since both methods are applied during fine-tuning, the computational demands are not a significant concern.

**Table 3:** Comparing ANAA with naive masking and other hidden representation augmentation methods.

**Model/downstream task**	**HF prediction (MDC)**	**HF prediction (MIMIC-IV)**
Transformer pre-trained on MLM	72.2 (2.5)	85.2 (1.1)
Transformer pre-trained on MLM+ Naive masking	70.00 (1.5)	85.1 (0.7)
Transformer pre-trained on MLM+ DropAttention	69.7 (1.1)	84.9 (1.3)
Transformer pre-trained on MLM+ NEFTune (α = 5)	73.6 (3.2)	85.2 (0.7)
Transformer pre-trained on MLM+ NEFTune (α = 10)	73.1 (1.7)	85.5 (0.4)
Transformer pre-trained on MLM+ noise in the feedforward (α = 5)	73.7 (2.2)	85.0 (1.2)
Transformer pre-trained on MLM+ noise in the feedforward (α = 10)	72.5 (4.4)	84.5 (0.8)
Transformer pre-trained on MLM+ ANAA	**74.5 (2.9)**	**87.2 (0.4)**

The table shows the average AUC values (%) and standard deviation across HF prediction tasks on the MDC and MIMIC-IV datasets. Boldface indicates the best-performing model.

### 5.7 VS naive masking

Randomly masking the attention score matrix during training can be seen as an extreme form of RNA augmentation. Instead of adding normal noise to perturb relationships between events in a sequence, naive masking directly disrupts these relationships by summing each element with 0 or −*A*_*h*_*i, j*__, effectively breaking the connections between tokens. We compared our method with naive self-attention masking, as described by (Wu et al. 2023), which introduces a bias in the structure of self-attentions:


(12) $$\begin{array}{c} A_{h}=\text{softmax}\left(\frac{Q_{h}K_{h}^{T}}{\sqrt{d_{k}}}+M\right),\;M\in {\left\{0,-\infty \right\}}^{N\times N}, \end{array}$$

where *M*_*i, j*_ = −∞ with *p* = 0.2, optimized based on performance on the fine-tuning dataset. We extended it to DropAttention (Zehui et al., 2019), which expands the mask with a span length ω and we set ω = Kernel size. However, neither naive masking nor DropAttention improved the performance of the pre-trained Transformer for HF prediction on the MDC and MIMIC-IV datasets. Instead, these methods only increased the number of training iterations required for convergence (see Table 3). While these techniques can help mitigate overfitting, their overly aggressive regularization often disrupts critical dependencies within sequences, leading to unstable training and poorer overall performance, especially on complex healthcare prediction tasks. In contrast, ANAA introduces controlled perturbations that balance the attention distribution and prevent over-reliance on specific patterns, thereby preserving essential relationships in the data and promoting more robust and effective representations (see Supplementary material Section 1.7 for a justification of ANAA as a structured variant of dropout).

### 5.8 Effect of ANAA on self-attention behavior

Analyzing self-attention weights and attention score matrices can highlight how Transformers prioritize relationships between events, shedding light on their internal logic and behavior (Clark et al., 2019; Kovaleva et al., 2019; Hao et al., 2021). To assess the effect of ANAA and compare it with normal noise injection (RNA), we analyzed attention score distributions in models fine-tuned on all downstream tasks.

We plotted histograms of attention scores across all heads and samples from the test split, scaling each head's scores to the [0, 1] range (Figure 4). In the bottom row of the figure, we observe that attention scores from the fine-tuned vanilla Transformer tend to cluster near 0 or 1, forming a near-binary (binomial-like) distribution. This pattern suggests overconfidence and limited exploration of dependencies across tokens.

In contrast, the middle row shows that RNA—injecting Gaussian noise during training—broadens the distribution, encouraging attention heads to explore more diverse and weaker connections. This leads to overlapping attention patterns and increased representation diversity. A mathematical explanation for this phenomenon is provided in Supplementary material Section 1.5.

The top row demonstrates the effect of ANAA, which combines noise injection with Gaussian smoothing. This operation retains the diversity introduced by noise while stabilizing the attention pattern, restoring smoother and more informative distributions. The smoothing step dampens extreme noise while allowing the model to refine its exploration of differnt interactions.

**Figure 5:**
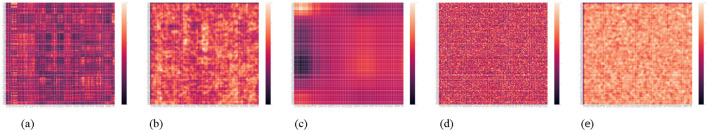
Comparing the impact of ANAA on the self-attention score weights for five fine-tuned models on HF prediction on the MDC dataset for a specific test sample. Here, the attention scores are scaled within 0 and 1. **(a)** Transformer. **(b)** Transformer + ANAA. **(c)** Pre-trained Transformer. **(d)** Pre-trained Transformer + RNA. **(e)** Pre-trained Transformer + ANAA.

To further investigate, we visualized the attention score matrices from models fine-tuned on a representative test sample from the HF prediction task on the MDC dataset (Figure 5). Comparing the original and smoothed attention scores, we observe that ANAA promotes broader attention coverage, with activation scores scaled to the [0, 1] range. Figure 5 illustrates an attention head from the first layer, confirming that ANAA leads to more distributed attention patterns. Additional examples from the MIMIC-IV dataset are provided in the Supplementary material Section 1.10.

However, it is important to note that the heat-maps in Figures 4, 5 are intended as qualitative diagnostics of how ANAA redistributes attention–not to explain the model's decisions. As shown in prior work, attention weights can often be manipulated without affecting model outputs, meaning they are not a reliable source of explanation (Hao et al., 2021; Jain and Wallace, 2019; Serrano and Smith, 2019). We therefore interpret these visualizations only as evidence that ANAA breaks the near-binary pattern observed in the baseline model; attributing clinical relevance to specific codes and specific codes with each other in this context would require dedicated methods such as Integrated Gradients (Sundararajan et al., 2017) and can be investigated further in future work.

#### 5.8.1 Effect of ANAA on the receptive field

**Figure 6:**
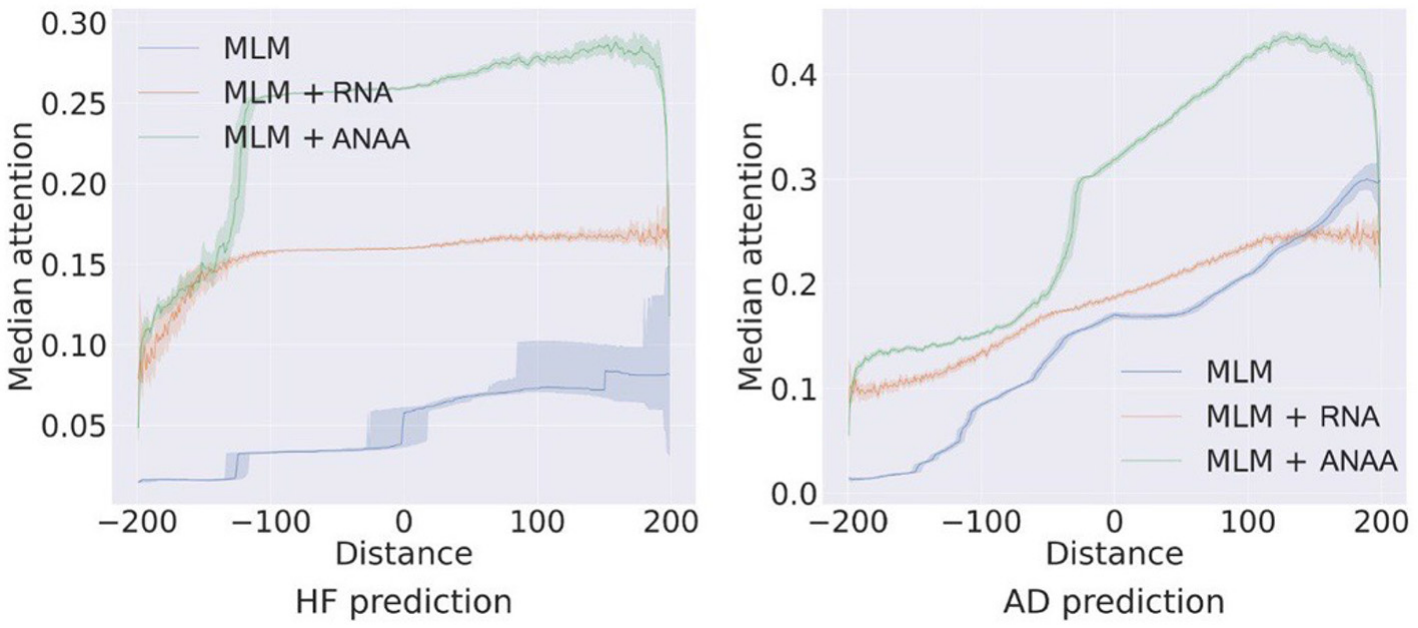
Impact of ANAA on the receptive field of the self-attentions for HF and AD prediction on the MDC dataset.

The self-attention mechanism is designed to capture both long and short-range dependencies effectively. To quantitatively assess the impact of RNA and ANAA on the receptive field, we plot the median values of attention score matrix *A*_*h*_ for each event with respect to all previous and subsequent events (*i*−*j, A*_*h*_*i, j*__) -*i, j* are positions of *e*_*i*_, *e*_*j*_ in the sequence of events-across all test samples for HF and AD predictions on the MDC (Figure 6). Transformers pre-trained on MLM typically allocate more attention weight to recent events, often in a monotonous fashion. Incorporating RNA regularization reduces the steepness of this attention distribution, allowing events to receive more balanced attention, not solely based on their proximity to recent events. Ultimately, applying ANAA, preserves the benefits of RNA by providing a more equal distribution of attention within a local neighborhood, while simultaneously reducing the emphasis on very distant past events.

## 6 Discussion

This study demonstrates that ANAA—a simple two-step fine-tuning augmentation—consistently enhances the discriminative performance of pre-trained Transformers on longitudinal EHR data, without altering their architecture. Compared to the hidden representation augmentation and a range of established regularizers, it yields superior results over vanilla fine-tuning. ANAA produced consistent AUC gains on HF and AD prediction tasks in two different EHR corpora (MDC and MIMIC-IV). On HF prediction, for example, the MLM-pre-trained baseline rose from 72.2 to 74.5 AUC on MDC and from 85.2 to 87.2 AUC on MIMIC-IV after applying ANAA. These gains persisted even under label-scarce conditions, maintaining ~3 percentage-point improvements. These findings suggest that judicious noise injection at the level of self-attention—followed by controlled Gaussian smoothing—can encourage pre-trained transformers to explore and learn more robust, generalizable patterns.

We further investigated that Transformers pre-trained via MLM—while typically outperforming models without pre-training—can exhibit overconfident, sparse attention patterns during fine-tuning. Attention histograms reveal that conventional fine-tuning drives many heads toward almost binary (0/1) weights, indicating over-confident, brittle dependencies. ANAA counteracts this by injecting adaptive Gaussian noise, which broadens the attention distribution and encourages heads to sample a richer set of relational cues. The subsequent smoothing step restores coherent structure. As shown analytically in Supplementary material Section 1.5, this mechanism effectively acts as a variance-scaled, shifting the attention score distribution from deterministic and binary to probabilistic and continuous, to explore alternative dependencies.

Compared to other augmentation methods such as NEFTune (Jain et al., 2023) and HyPe (Yuan et al., 2022)—which add noise in the embedding or feed-forward layers—ANAA achieves larger and more consistent performance gains. In contrast, naive attention masking or DropAttention (Wu et al., 2023; Zehui et al., 2019) degraded results. This highlights the importance of *where* noise is injected: perturbing the self-attention scores—the core mechanism for modeling token interactions—yields greater benefit than altering downstream representations.

While ANAA consistently improves performance across the two studied EHR datasets, several caveats remain. First, all experiments were conducted on structured, diagnosis- and medication-coded timelines (MIMIC-IV and MDC); Although our experiments focus on a standard Transformer encoder for clarity and control, ANAA is modular by design and can be integrated into other clinical Transformer models such as BEHRT (Li et al., 2020), Med-BERT (Rasmy et al., 2021), or Hi-BEHRT (Li et al., 2022); exploring such integrations is a promising direction for future work. More broadly, how well ANAA generalizes to other data modalities –such as free text, imaging, or genomics –and to models pre-trained with alternative objectives such as contrastive learning (e.g., BYOL; Grill et al., 2020) also remains to be explored. Second, ANAA introduces additional hyperparameters. Although the sensitivity analysis in Supplementary Table S3 suggests the method is robust across a range of values, some tuning is still required. Third, the computational overhead introduced by noise injection and smoothing increases both memory usage and training time, which may become a limitation for very long sequences or resource-constrained environments. Fourth, in settings with extremely low data regimes or highly unbalanced labels, ANAA's implicit Augmentation provides some benefit but is not sufficient on its own. Finally, the effect of model augmentations, like ANAA, on model interpretability warrants further study, particularly in safety-critical applications.

## 7 Conclusion

We introduced *Adaptive Noise-Augmented Attention* (ANAA), a lightweight and effective method for enhancing the fine-tuning of pre-trained Transformers. ANAA directly augments the self-attention scores with adaptive Gaussian noise and applies a smoothing convolution using a Gaussian kernel, encouraging the model to explore more diverse attention patterns while preserving critical dependencies.

We demonstrated that pre-trained Transformers, when fine-tuned on limited EHR datasets, often converge to overly sharp attention distributions—overfitting to local patterns and failing to capture broader contextual relationships. ANAA mitigates this by encouraging more diverse and stable attention distributions, leading to better generalization across tasks and data regimes. Extensive experiments on multiple clinical prediction tasks showed that ANAA consistently outperforms conventional regularization and hidden augmentation techniques.

ANAA offers a plug-and-play augmentation mechanism that operates entirely within the attention computation, requiring no modification to the model architecture or computational graph. This makes it particularly suitable for integration with existing pre-trained models.

## Data Availability

The MIMIC-IV dataset is publicly available from the PhysioNet repository [https://physionet.org/content/mimiciv/2.2/]. The Malmo Diet and Cancer Cohort data that support the findings of this study are not publicly available due to data access restrictions imposed by the Malmo Population-Based Cohorts Joint Database. However, the data are available from the corresponding author upon reasonable request and with permission from the Malmo Population-Based Cohorts Joint Database [https://www.malmo-kohorter.lu.se/malmo-cohorts].
