# Supplementary material for: Adaptive noise-augmented attention for enhancing Transformer fine-tuning on longitudinal medical data
Source: Front Artif Intell. 2025 Sep 17;8:1663484. doi: 10.3389/frai.2025.1663484 (PMC12484044; doi:10.3389/frai.2025.1663484)
Supplement: Supplementary file 1 [file Data_Sheet_1.pdf]

Supplementary Material

1 SUPPLEMENTARY MATERIAL

1.1 Implementation Details

The code compatible with the public MIMIC-IV dataset is provided in the Code Appendix.

The Transformer encoder was tuned on the MLM pretraining task based on the HF prediction results on the validation dataset. We experimented with different hyperparameter configurations, including the number of attention heads set to 1, 3, 5, and 8, hidden dimensions of 16, 36, and 64, and Transformer encoder layers set to 1, 4, and 8. The best-performing configuration consisted of five attention heads, a hidden dimension of 36, and a single Transformer encoder layer.

For MLM pretraining, we followed the approach in Poulain et al. (2022), where 15% of medical tokens were randomly selected for modification. Of these masked tokens, 80% were replaced with the `[MASK]` token, 10% were replaced with a randomly selected different medical token, and the remaining 10% were left unchanged.

1.1.1 Pretraining on MLM

For pretraining, we used the pretraining splits for each cohort as described in Section 1.2. The input sequence length was determined as the 0.7 percentile of the distribution of sequence lengths in the pretraining dataset, which resulted in 131 tokens for the MDC dataset and 65 tokens for the MIMIC-IV dataset. A sliding window approach with a stride of one was used to augment data during the MLM pretraining phase. This resulted in approximately 313,000 training samples and 33,000 validation samples for MDC, while MIMIC-IV contained around 1.245 million training samples and 140,000 validation samples. The vocabulary sizes were 1,675 for MDC and 2,338 for MIMIC-IV. The models were pre-trained for 50 epochs, after which the training and validation losses plateaued, as shown in Figure S1.

1.1.2 Training and Fine-Tuning on Downstream Task Prediction

Hyperparameters were optimized for HF prediction using the MLM pre-trained Transformer without augmentation. The same values were then used to evaluate the effect of adding ANAA, without additional tuning. For fine-tuning, token representations were aggregated using a GRU layer before being fed into a classifier. The optimizer and the learning rate and coefficient, warm up steps and dropout were optimized from [SGD, Adam, AdamW], [1e-5,2e-5,5e-5,7e-5], co=[1,.95,.9,.8] and warmup=[0,.05,.1,.2]*total steps and dropout=[0, .1, .2,.4].The model was trained using the Adam (for 1 layer and ADAMW for 4 and 8 layer) optimizer with a layer-wise learning rate decay coefficient of 0.9 and an initial learning rate of 6×10^{-5}. The input sequence length was set to 200 medical codes.

1.1.3 Cross-Validation

For model evaluation, five-fold cross-validation was conducted. The dataset was divided into five folds, and the model was fine-tuned on four folds while early stopping was applied using the remaining fold. This process was repeated five times with different validation sets, and the reported results include the mean and standard deviation of the AUC on the unseen test dataset.

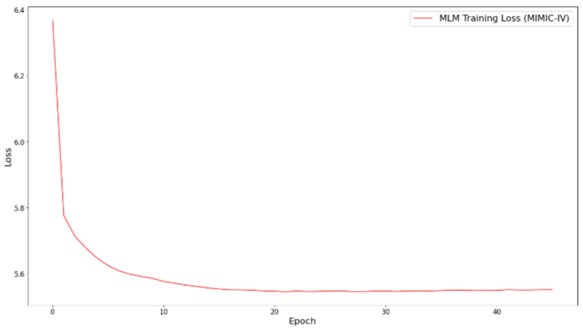

(a) MLM training loss for MIMIC-IV

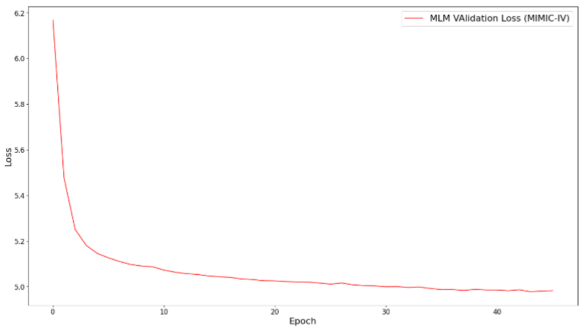

(b) MLM validation loss for MIMIC-IV

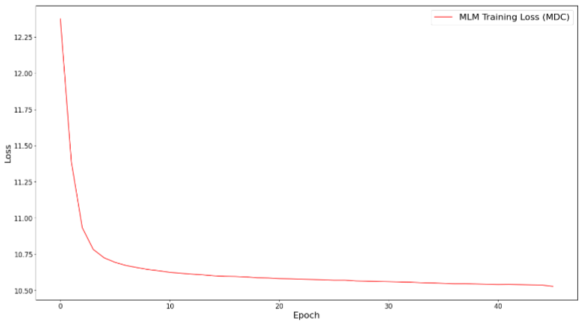

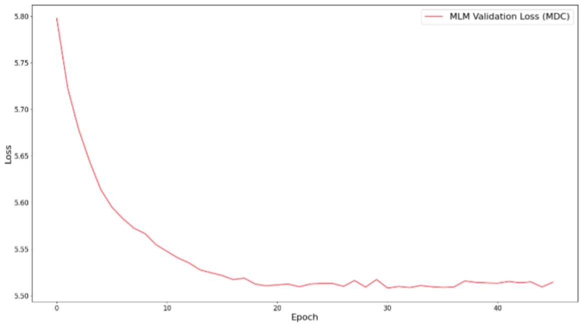

Figure S1: MLM training and validation loss for MIMIC-IV and MDC datasets during training.

1.2 Dataset specifications

We used medical data from two sources: the Medical Information Mart for Intensive Care IV (MIMIC-IV) Johnson et al. (2020) hosp module, and the Malmö Diet and Cancer Cohort (MDC) Berglund et al. (1993) dataset, approved by the Ethics Review Board of Sweden (Dnr 2023-00503-01). Each EHR trajectory represents a sequence of events of temporal structured health data. The MIMIC-IV hosp module is a comprehensive collection of inpatient EHR trajectories, containing approximately 173,000 patient records documented during 407,000 visits spanning from 2008 to 2019. This dataset includes a total of 10.6 million medical codes representing diagnoses and medications.

On the other hand, the MDC dataset originates from a prospective cohort study conducted in Sweden. It consists of around 30,000 individuals residing in Malmö between 1991 and 1996, with records of both inpatient and outpatient visits spanning from 1992 to 2020, resulting in a total of 531,000 visits. While the MDC dataset has fewer overall samples, it provides a more extensive patient history, with an average of 257 codes per patient compared to MIMIC-IV's 61.

Both datasets use the International Statistical Classification of Diseases and Related Health Problems (ICD) and Anatomical Therapeutic Chemical Code (ATC) for disease and medication classification, respectively, in a hierarchical format.

To facilitate our self-supervised pre-training, supervised fine-tuning, and final testing, we partitioned the extracted cohort randomly into three subsets: 70%, 20%, and 10%, respectively. Despite being characterized by extensive sparsity, preprocessing resulted in 2,195 unique ICD-9 and 137 unique ATC-5 codes for the MIMIC-IV dataset and 1,558 unique ICD10 and 111 unique ATC-5 codes for the MDC dataset.

Figure S2 presents boxplots (excluding outliers) for the number of visits and number of medical codes per patient in the MDC and MIMIC-IV datasets.

Table S1. MIMIC-IV dataset summary statistics.

	Pre-training	Fine-tuning	Test	Total
Patients	121 k	36 k	16 k	173 k
Visits	285 k	86 k	37 k	408 k
Medical codes	7.451 M	2.234 M	0.937 M	10.622 M

Table S2. MDC dataset summary statistics.

	Pre-training	Fine-tuning	Test	Total
Patients	21 k	6 k	3 k	30 k
Visits	373 k	107 k	52 k	531 k
Medical codes	5.339 M	1.554 M	0.741 M	7.634 M

1.3 Effect of Varying σ_{eh}

Table S3 shows the AUC scores on the validation split for each downstream task using a range of σ_{eh} values and their corresponding Gaussian kernel sizes. We probe the region where model performance is most sensitive with fine resolution (sequence length to kernel size ratio), while avoiding an exhaustive—and prohibitively costly—grid search across the entire range. Overall, ANAA shows robust performance across different σ_{eh} values, with optimal results achieved consistently within each dataset. Notably, for the MDC dataset, performance on the HF prediction task is more sensitive to changes in σ_{eh}, while other tasks remain relatively stable.

Table S3. Effect of different σ_{eh} values (and the corresponding kernel sizes) on validation-split AUC for four downstream tasks.

**Task / σ_{eh}**	**0.3 (k=2)**	**0.6 (k=4)**	**1.0 (k=6)**	**1.5 (k=10)**	**3.1 (k=20)**	**6.5 (k=40)**	**13.4 (k=80)**
HF prediction (MDC)	0.791	0.790	**0.841**	0.822	0.771	0.792	0.811
AD prediction (MDC)	0.800	0.788	**0.804**	0.782	0.791	0.803	0.796
HF prediction (MIMIC-IV)	**0.923**	0.910	0.914	0.914	0.901	0.910	0.918
PLS prediction (MIMIC-IV)	**0.603**	0.598	0.591	0.603	0.599	0.588	0.597

1.4 Data availability

The MIMIC-IV data is available on `https://physionet.org/content/mimiciv/2.2/`. The MDC dataset is available upon application and with permission of the Malmo Population-Based Cohorts Joint Database `https://www.malmo-kohorter.lu.se/malmo-cohorts`

1.5 Mathematical Justification: Impact of Normal Noise on Self-Attention distribution

1.5.1 Effect of Noise on Self-Attention

As shown in the bottom row of Fig. 4 in the main manuscript, attention scores in pre-trained Transformers often converge to extreme values—either close to 0 or 1—after fine-tuning. This suggests that the model makes sharp, deterministic decisions regarding which tokens to attend to. To introduce stochasticity and encourage more flexible learning of dependencies, we inject Gaussian noise into the attention scores during training. In this section, we analyze how this perturbation affects the distribution of attention scores.

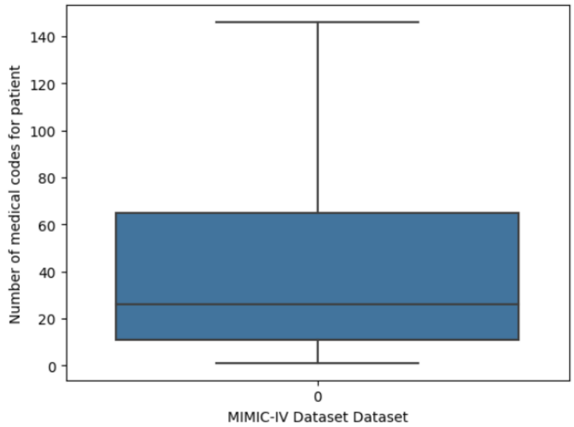

(a) Number of medical codes per patient

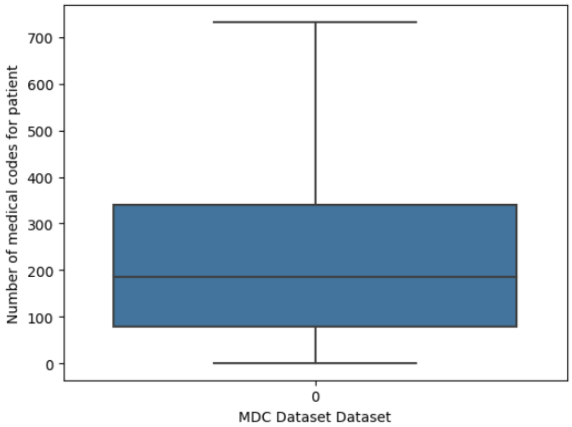

(b) Number of visits per patient

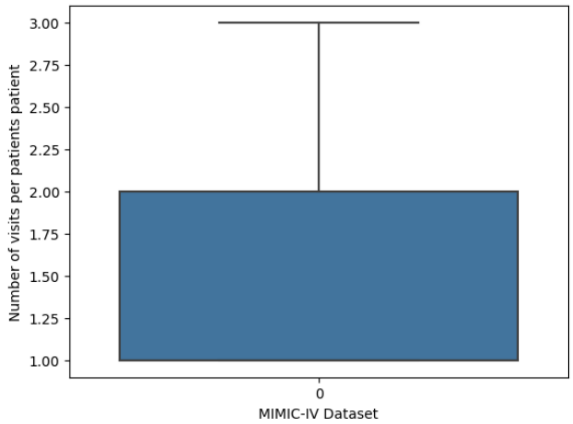

(c) Number of medical codes per patient

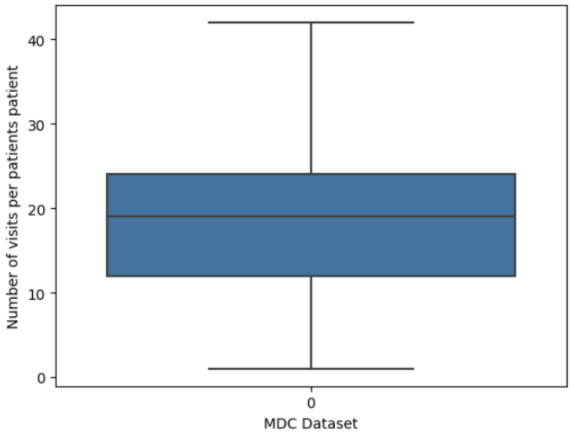

(d) Number of visits per patient

Figure S2: Box plot showing the distribution of the number of medical codes and visits per patient in the MIMIC-IV and MDC datasets.

1.5.2 Original Distribution of Attention Scores

From empirical observations in Fig. 4 in the main manuscript, we approximate the attention score distribution as bimodal—concentrated at 0 and 1. This can be modeled as a mixture of two Dirac delta functions:

$$p(A_h) = \alpha\delta(A_h - 0) + (1 - \alpha)\delta(A_h - 1)$$

where α is the weight for the peak at 0, and $1 - \alpha$ is the weight for the peak at 1.

The mean and variance of this distribution are:

$$\mathbb{E}[A_h] = (1 - \alpha)$$
$$\mathbb{E}[A_h^2] = (1 - \alpha)$$

Thus, the variance of the attention scores is:

$$\mathrm{Var}(A_h) = \mathbb{E}[A_h^2] - (\mathbb{E}[A_h])^2 = (1-\alpha) - (1-\alpha)^2 = \alpha(1-\alpha)$$

This gives the standard deviation:

$$\mathrm{std}(A_h) = \sqrt{\alpha(1-\alpha)}$$

1.5.3 Effect of Gaussian Noise Injection

We inject Gaussian noise into the attention scores during training:

$$A'_h = A_h + \epsilon \quad \text{where} \quad \epsilon \sim \mathcal{N}(\mu, \mathrm{std}(A_h)^2)$$

The noise ϵ is drawn from a normal distribution with μ mean and variance $\mathrm{std}(A_h)^2 = \alpha(1-\alpha)$.

Thus, the perturbed distribution becomes:

$$A'_h = \begin{cases} \mathcal{N}(\mu, \alpha(1-\alpha)), & \text{if } A_h = 0, \\ \mathcal{N}(1+\mu, \alpha(1-\alpha)), & \text{if } A_h = 1. \end{cases}$$

Thus, the original binary peaks at 0 and 1 are smoothed into overlapping Gaussian centered at μ and $1+\mu$ respectively.

1.5.4 Effect on the Distribution of Attention Scores

This transformation shifts the attention distribution from discrete to continuous, promoting diversity in attended interactions:

- When $A_h = 0$, the perturbed attention score A'_h will follow a normal distribution centered at μ .
- When $A_h = 1$, the perturbed attention score A'_h will follow a normal distribution centered at $1+\mu$.

As shown in the middle row of Fig. 4 in the main manuscript, this noise increases overlap between previously distinct peaks. When rescaled between 0 and 1, the distribution appears more centralized and continuous. Notably, the variance is maximized when $\alpha = \frac{1}{2}$, which results in the greatest overlap and diversity.

1.5.5 Conclusion

Injecting Gaussian noise shifts the attention score distribution from deterministic and binary to probabilistic and continuous, encouraging the model to explore alternative dependencies. The overlap of noised distributions allows the model to escape rigid patterns and better capture complex, context-dependent relationships. Crucially, our use of an adaptive standard deviation based on the attention scores allows the model to modulate this behavior based on the context, facilitating better generalization and diversity across heads and layers.

1.5.6 Regularization and Robustness

Beyond promoting attention diversity, noise injection acts as a regularizer with several benefits:

- It prevents overfitting to strong patterns by injecting uncertainty.
- It encourages attention to low-probability events.
- It increases the variance of attention distributions, enabling the model to attend to underrepresented interactions.
- It promotes exploration and robustness

These effects support improved generalization in data-scarce environments and more expressive modeling.

1.6 Justification: Impact of Gaussian Smoothing

The smoothing operation is used to adjust the added noise.

1.6.1 Effect of Gaussian Convolution on Attention Matrix

The convolution operation:

$$A''_h = (A_h + \mathcal{N}(\mu, \sigma^2_{GN})) * n_{\sigma_{eh}} \tag{S1}$$

performs localized averaging of the noisy attention matrix $A_h + \mathcal{N}(\mu, \sigma^2_{GN})$, where:

$$A''_h(i,j) = \sum_{m=1}^{k}\sum_{n=1}^{k} A'_h(i-m, j-n) \cdot n_{\sigma_{eh}}[m,n] \tag{S2}$$

This operation results in three key effects:

1. Noise Reduction: Independent Gaussian noise can introduce high-frequency fluctuations. Convolving with a Gaussian kernel acts as a low-pass filter, reducing such variance.
2. parsity Reduction: In the original attention, many values may be near-zero, creating sparsity. Smoothing fills in values by interpolating between neighbors, producing more continuous transitions.
3. Robust Contextualization: Original patterns are preserved and extended through smoothing

1.7 Justification for ANAA: A Comparison with Dropout

ANAA can be justified by drawing parallels with dropout regularization, viewing ANAA as an "adaptive" extension of it. Dropout works by randomly setting some of the activations to zero, effectively disconnecting certain nodes during training. This prevents the model from becoming overly dependent on specific neurons and encourages a more robust and generalized representation.

Mathematically, for each attention head A_h, dropout can be seen as applying a mask M (where M is a Bernoulli distribution), resulting in the modified attention head $A'_h = M \cdot A_h$. In contrast, ANAA applies a more nuanced adjustment:

$$A_h = A_h + \epsilon \sim \mathcal{N}(\mu, \sigma^2) = \left(1 + \frac{\epsilon}{A_h}\right) A_h = P \cdot A_h \tag{S3}$$

Here, $P = \left(1 + \frac{\epsilon}{A_h}\right)$ acts as an adaptive perturbation factor. Instead of completely severing connections between tokens (as in dropout), ANAA adjusts the attention weights by either amplifying or diminishing the

focus between two events. This approach maintains the relationships within the data while still introducing variability.

The random perturbation from ANAA forces the attention mechanism to avoid over-reliance on specific patterns by continually adjusting the attention distribution. Consequently, ANAA can be seen as a form of ensemble learning, where each perturbation offers a different perspective on the data. This effectively trains multiple versions of the model in parallel, each slightly varied due to the noise, leading to a more robust and generalized final model.

1.8 Performance boost on data insufficiency

Table S4 presents the numerical results corresponding to the data insufficiency section.

Table S4. Effect of incorporating ANAA into the pre-trained Transformer on AUC performance value of HF prediction across various fine-tunning sample sizes on the test dataset in MIMICIV and the MDC

Model–dataset / fine-tuning	10%	20%	50%	100%
MLM–MDC	0.500 (0.000)	0.637 (0.053)	0.710 (0.030)	0.722 (0.025)
MLM+ANAA–MDC	0.500 (0.000)	0.628 (0.041)	0.738 (0.012)	0.745 (0.029)
MLM–MIMIC-IV	0.800 (0.003)	0.819 (0.011)	0.834 (0.005)	0.852 (0.011)
MLM+ANAA–MIMIC-IV	0.835 (0.023)	0.849 (0.013)	0.860 (0.007)	0.872 (0.004)

1.9 Computational Complexity and Scalability

ANAA involves two primary operations per attention head: adding iid normal noise to an $n \times n$ attention score matrix and convolving the result with a Gaussian filter. Therefore, the computational complexity of ANAA can be expressed as:

$$O(\text{ANAA}) = O(\text{addition of } n \times n \text{ matrix}) + O(\text{convolution})$$

$$O(\text{ANAA}) = O(n^2) + O(k^2 \times n^2) = O(n^2) + O(n^2) = O(n^2)$$

Here, n represents the input length (where $n = 200$ in our case), and k is the kernel size ($k \ll n$). Since the noise addition and convolution operations are only performed during the fine-tuning phase—where the number of samples is significantly smaller compared to pre-training—ANAA introduces minimal scalability limitations.

1.10 Effect of ANAA on self-attention behavior on the MIMIC-IV dataset

Figure S3 shows the effect of augmenting pre-trained Transformers with RNA and ANAA on a specific sample on the MIMIC-IV dataset.

1.11 Baseline Models

The baselines in our study were selected based on prior research and practical considerations for modeling temporal health data. The following models were used for comparison:

- **Logistic Regression (LR)**
- **Random Forest (RF)**
- **Multilayer Perceptron (MLP)**
- **Bidirectional GRU (Bi-GRU)**

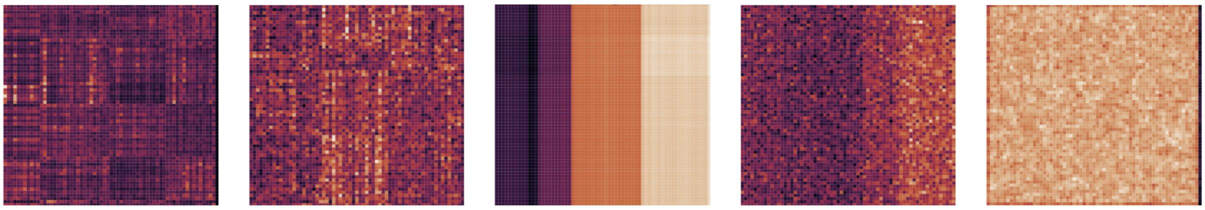

(a) Transformer (b) Transformer+ANAA (c) Pre-trained Transformer (d) Pre-trained Transformer+RNA (e) Pre-trained Transformer+ANAA

Figure S3: Attention-weight heatmaps for five fine-tuned models on the HF prediction task in MIMIC-IV (one representative sample). The colour scale is normalised independently in each panel.

- **Transformer (scratch)**: A Transformer encoder trained from scratch using multi-head attention followed by a classification feedforward head.
- **Pretrained Transformer (MLM)**: A Transformer encoder pretrained on MLM and fine-tuned on downstream tasks, following approaches such as BEHRT, Med-BERT, and others (Rasmy et al., 2021; Li et al., 2020; Meng et al., 2021).

For LR, RF, and MLP, each visit was encoded as a multi-hot vector and aggregated via summation across visits. These baselines allow us to benchmark the performance of ANAA against both classical machine learning models and modern Transformer-based architectures. Training and fine-tuning hyperparameters for each model are provided in Appendix 1.1. Table S5 shows the results.

Table S5. Average AUC (%) and standard deviation of different baseline methods for HF prediction, AD prediction, and PLS prediction on the test datasets.

Model / Task	HF (MDC)	AD (MDC)	HF (MIMIC-IV)	PLS (MIMIC-IV)
Logistic regression	62.4 (1.1)	56.4 (1.1)	83.9 (1.2)	54.2 (0.4)
Random forest	60.7 (0.5)	51.8 (0.3)	77.2 (2.3)	51.1 (0.3)
MLP	67.9 (3.0)	68.0 (1.5)	85.2 (0.3)	59.3 (1.9)
Bi-GRU	62.3 (1.2)	60.4 (1.1)	86.5 (1.2)	55.9 (1.0)

REFERENCES

Berglund, G., Elmståhl, S., Janzon, L., and Larsson, S. (1993). The malmo diet and cancer study. design and feasibility. *Journal of internal medicine* 233, 45–51

Johnson, A., Bulgarelli, L., Pollard, T., Horng, S., Celi, L. A., and Mark, R. (2020). Mimic-iv. *PhysioNet. Available online at: https://physionet. org/content/mimiciv/1.0/(accessed August 23, 2021)*

Li, Y., Rao, S., Solares, J. R. A., Hassaine, A., Ramakrishnan, R., Canoy, D., et al. (2020). Behrt: transformer for electronic health records. *Scientific reports* 10, 7155

Meng, Y., Speier, W., Ong, M. K., and Arnold, C. W. (2021). Bidirectional representation learning from transformers using multimodal electronic health record data to predict depression. *IEEE journal of biomedical and health informatics* 25, 3121–3129

Poulain, R., Gupta, M., and Beheshti, R. (2022). Few-shot learning with semi-supervised transformers for electronic health records. In *Machine Learning for Healthcare Conference* (PMLR), 853–873

Rasmy, L., Xiang, Y., Xie, Z., Tao, C., and Zhi, D. (2021). Med-bert: pretrained contextualized embeddings on large-scale structured electronic health records for disease prediction. *NPJ digital medicine* 4, 86
